# Navigating the complexities of teaching rounds: balancing educational and patient-centered objectives

**DOI:** 10.3389/fmed.2025.1615532

**Published:** 2025-06-26

**Authors:** Yang Li, Lu Lu, Yanming Yin, Aijun Wang, Denghui Huang, Wenjiang Yang

**Affiliations:** ^1^Science and Education Section, Wenshang County People's Hospital, Jining, Shandong, China; ^2^English Research Group, Wenshang County No. 1 Middle School, Jining, Shandong, China; ^3^Medical Department, Wenshang County People's Hospital, Jining, Shandong, China; ^4^Wenshang County People's Hospital, Jining, Shandong, China; ^5^Urinary Surgery, Wenshang County People's Hospital, Jining, Shandong, China; ^6^Department of Surgery, Wenshang County People's Hospital, Jining, Shandong, China

**Keywords:** hospital education department, medical education, educational technology, patient-centered care, teaching rounds

## Abstract

**Background:**

Teaching rounds are a fundamental component of medical education, offering essential clinical learning opportunities for students while ensuring high-quality care for patients. Despite their importance, the psychological and emotional experiences of participants—attending physicians, interns, and patients—during these rounds remain underexplored. This study addresses this gap by examining the impact of patient presence on the learning environment, focusing on three key themes: psychological comfort and anxiety, cognitive load management, and emotional engagement and detachment.

**Material and methods:**

A design-based research approach was employed, conducted in a surgical ward affiliated with a medical college. The study involved 40 participants, including attending physicians, interns, and patients. Data were collected through 40 in-depth interviews, with analysis focusing on the experiences and perspectives of all parties involved.

**Results:**

The findings reveal that transparency in communication is crucial for building trust but can also induce anxiety among both trainees and physicians due to the scrutiny of their actions. Managing cognitive load was identified as a significant challenge, particularly for interns who must balance learning with patient interaction. Emotional engagement is vital for effective patient care but must be carefully balanced with professional detachment to maintain clinical objectivity.

**Conclusion:**

This study contributes to the understanding of the complex dynamics in teaching rounds, emphasizing the need for strategies that balance educational goals with patient-centered care. The insights gained offer valuable guidance for enhancing the learning environment in medical education.

## 1 Introduction

Teaching rounds are fundamental to medical education, providing students essential clinical learning experiences while ensuring the delivery of high-quality patient care (1). However, the intricate dynamics within teaching rounds, involving attending physicians, interns, and patients, introduce challenges that can affect both educational outcomes and patient care. Despite the critical role of teaching rounds in medical training, the psychological and emotional experiences of participants during these sessions remain insufficiently studied.

Recent studies underscore the significance of understanding these experiences, especially in terms of psychological comfort, cognitive load, and emotional engagement (2). Psychological comfort, for instance, is closely linked to transparency in communication (3). Transparency builds trust between patients and healthcare providers and alleviates trainee anxiety in unpredictable clinical environments (4). However, this transparency can also increase anxiety for both trainees and attending physicians, who may feel scrutinized by patients and colleagues (5).

Cognitive load management is another critical factor that influences the effectiveness of teaching rounds (6). Attending physicians must balance the dual responsibilities of providing clinically relevant information and ensuring that this information is comprehensible to both trainees and patients. The cognitive demands on trainees are significant, as they are required to absorb complex information, engage with patients, and simultaneously learn from the experience. Patients, on the other hand, may struggle with understanding medical jargon, which can lead to feelings of alienation and confusion (7).

Emotional engagement and detachment add further complexity to the teaching dynamic. While empathetic engagement is essential for establishing rapport with patients and improving the quality of care, it must be carefully balanced with professional detachment to maintain objective decision-making (8). Both trainees and attending physicians must manage this delicate balance, as excessive emotional involvement can compromise clinical judgment, whereas excessive detachment may be perceived by patients as a lack of care.

In this study, we aim to explore how the presence of patients during teaching rounds affects the learning environment and to gather insights from all participants—interns, attending physicians, and patients. We focus on three main themes: psychological comfort and anxiety, cognitive load management, and emotional engagement and detachment. Understanding these themes is crucial for developing strategies that enhance the learning environment in medical education, ensuring it is conducive to both effective learning and patient care.

This study was conducted in a surgical ward of a hospital affiliated with a medical college, where patients were actively involved in all teaching and learning activities. The interventions included having interns present the entire case to the attending physician in the presence of the patient, followed by case discussions also conducted in the patient’s presence. The findings from this study are anticipated to inform the ongoing refinement of teaching strategies that emphasize both educational effectiveness and patient-centered care.

## 2 Methods

### 2.1 Subjects

The study involved patients, learners (interns), attending physicians, and researchers. The research team held regular meetings to refine research questions, develop interview guides, and discuss and analyze the findings. Interviews were conducted by three researchers with distinct backgrounds: A lead physician (15 years clinical/teaching), an academic researcher (Medical Education), a Patient Representative (Hospital Patient Advisory Board). All interviewers received practical training in conducting semi-structured interviews. After each interview was recorded, the relevant survey results were incorporated into the interview guide to enrich the subsequent data collection process.

### 2.2 Experimental design

A design-based research method used to guide our research. Interview guides are planned, tested and refined. The question for the study was to explore how the presence of patients affects the learning environment and to collect evaluations from all participants - learners (interns), attending physicians, and most importantly, patients. We applied the conceptual framework of agency, the degree to which people feel free or constrained in a given situation, as a framework for our design and conduct of our documentation. Among them, the feelings expressed by the patients about the scene, as well as the feedback of the medical activities of the interns and attending physicians, were recorded.

### 2.3 Settings

The study was conducted in a surgical ward at Wenshang County People’s Hospital, Shandong, China – a tertiary teaching hospital affiliated with Jining Medical College. This setting reflects hierarchical medical education norms common in East Asia, where attending physicians hold significant authority. Patient inclusion protocols emphasized cultural considerations (e.g., family involvement in consent, deference to physicians). These contextual factors may limit direct transferability to decentralized Western systems but offer insights for similar hierarchical settings.

### 2.4 Intervention

The time span from the intervention to the interviews is as follows:

Patient interview: typically scheduled 1–2 days after the clinical teaching session to ensure the patient’s memory of the intervention remains fresh.
Intern interview: usually conducted 1–2 days after the patient interview, allowing the intern time to reflect on their performance and experience during the intervention.
Attending physician interview: generally arranged 1–2 days after the intern interview, providing the attending physician with an opportunity to assess the overall effectiveness of the intervention and the intern’s performance.

Overall, the time span from the initiation of the intervention to the completion of all interviews typically ranges from 1 to 2 weeks. This scheduling is designed to balance the timeliness and accuracy of feedback from all parties involved.

The attending physician conducts one to two patient instructional rounds each week. The study’s interventions involved having interns present the entire case to the attending physician in the presence of the patient. Additionally, all subsequent case discussions were conducted in front of the patient. Typically, the majority of discussions related to the case presentation occur in the classroom. Following the clinical instruction, we conducted interviews with each participant using interview guides specifically tailored for patients, interns, and attending physicians. The post-intervention interviews were carried out in three stages: (1) an interview with the patient after the clinical teaching session, (2) an interview with the interns, and (3) an interview with the attending physician.

### 2.5 Participants

In our study, the sample size was determined based on the principle of data saturation. We conducted 40 in-depth interviews, and during the analysis, we observed that as the interviews progressed, the emergence of new information and themes gradually diminished, eventually reaching data saturation. We also considered the sample size ranges recommended in similar studies and, taking into account practical resources and time constraints, determined the appropriate sample size. Additionally, we ensured participant diversity to enhance the representativeness and comprehensiveness of the research findings.

The study was approved by the hospital’s ethics committee. The informed consent was obtained from all subjects. Appropriate ethical aspects have been followed in all phases of the study, according to the Helsinki Declaration. All participants were informed about the study and its purposes; voluntary participation; data confidentiality, use, and processing; data protection statement; and contact details. The participants did not receive any rewards in return of participation. We recruited three types of participants: attending physicians, learners (interns), and patients along with their families, with a total sample size of 40 participants. The attending physicians included 4 senior physicians with extensive clinical experience. The group of learners consisted of 18 clinical medicine interns. The patient group comprised 18 randomly selected inpatients from the ward. Prior to the study, patients were informed that they could opt out at any time without impacting their clinical treatment to minimize any burden on them. Neither the attending physicians, the interns, nor the patients were made aware of the post-intervention interviews.

Patients for teaching rounds are selected based on the following criteria:

Educational objectives:
Diverse conditions: select patients with a range of conditions to provide learners with exposure to different diseases and management strategies.
Relevant learning points: choose cases that align with the session’s learning objectives, such as specific diagnostic challenges or treatment approaches.
Clinical skills: consider cases that facilitate the demonstration of essential clinical skills, decision-making processes, or critical thinking.

#### 2.5.1 Patient conditions

Stability: ensure the patient’s condition is relatively stable to allow teaching without compromising patient safety.

Complexity: include patients with complex or interesting cases that offer educational value, while avoiding overly complicated cases that may overwhelm learners.

#### 2.5.2 Patient consent

Informed Consent: always obtain explicit consent from patients or their guardians for participation in teaching rounds. Clearly explain the purpose, the individuals who will be present, and how their information will be used.

Voluntariness: emphasize that participation is voluntary and that they can withdraw consent at any time without affecting their care.

Privacy assurance: assure patients that their medical information will remain confidential and explain the measures taken to protect their privacy.

#### 2.5.3 Respect for patients

Comfort level: assess and respect the patient’s comfort with having multiple people involved in their care and discussing their condition in front of others.

Special considerations: be sensitive to issues such as terminal illness or severe emotional distress, and select cases accordingly.

#### 2.5.4 Logistical factors

Availability: ensure the patient will be available for the teaching session and is in an environment conducive to effective teaching, such as a ward room that can accommodate a group.

Timing: schedule rounds when the patient is likely to be most alert and receptive.

#### 2.5.5 Coordination with team

Consultation: collaborate with the patient’s primary care team to confirm that the patient’s condition is suitable for teaching and seek their input on case selection.

Feedback: incorporate feedback from previous sessions to continuously refine and improve the selection process.

#### 2.5.6 Ethical considerations

Patient autonomy: always prioritize the patient’s autonomy and ensure they are making an informed choice about their participation. Obtaining patient consent involved a detailed informed consent process where we provided patients with clear and comprehensive information about the teaching rounds, including its purpose, procedures, and potential impacts. We used simple language and provided written consent forms for patients to sign. Prior to the rounds, we held preparation meetings with patients to explain their role, address any concerns, and ensure their comfort. We also ensured strict confidentiality of patient information throughout the process. Ethical approval was obtained, and we have a feedback mechanism in place for patients to raise any issues or questions after their participation.

Professionalism: maintain professionalism and ensure that the presence of learners does not compromise the quality of patient care.

To ensure the inclusion and exclusion criteria for selecting hospitalized patients, we established clear guidelines. Inclusion criteria encompassed patients aged 18 and above who were hospitalized for at least 48 h and had a primary diagnosis relevant to our study. Exclusion criteria ruled out patients with severe cognitive impairments or those in the terminal stage of illness. To maintain fairness and randomness in the selection process, we used a random number generator to choose participants from the pool of eligible patients. Our sample aimed to capture diverse patient profiles, including variations in age, gender, and disease types, to ensure representativeness. We compared the characteristics of our sample with the overall patient population to confirm its diversity and representativeness. The implementation of these criteria was meticulously managed to ensure accurate and reliable data collection.

### 2.6 Data collection

The questions asked of the participants included the following three main areas: (1) around positive and negative experiences related to the learning environment; (2) Changes in the behavior and attitude of the medical team; (3) The overall impact on the learning environment. According to the research process based on interview guidelines, an iterative process of design, evaluation and redesign was followed. When interviews are completed, the interview guide is iteratively revised to include exploratory questions. If researchers find targeted ways to propose structured responses, or when previously formulated questions have not generated much discussion, we will revise these exploratory questions. Usually, after each set of interviews, the questions for the next interview are revised.

### 2.7 Data analysis

The research team comprised a lead physician, an academic researcher and full-time educator, and a patient researcher. Regular meetings were held to analyze and discuss the interviews. All co-investigators reviewed the transcribed interviews to understand the participants’ responses prior to analysis and to discuss and share emerging impressions. Subsequently, three team members independently coded the transcripts using NVivo™ version 12 (Doncaster, Australia), focusing on aspects generally considered important by the investigators, such as the depth of teaching, degree of learning, patient and participant engagement, and whether language was adapted to the intervention context. A deductive analysis approach was employed, utilizing an agent framework. We combined and categorized project-level codes into theoretically informed schema codes. These pattern codes were further refined through an iterative analysis process, with input from all co-investigators. Qualitative frequency analysis was performed post-interview to quantify recurring patterns. After a series of team meetings, where coding differences were discussed and negotiated, the research team reached a consensus.

Our study incorporated several strategies aligned with qualitative research standards:

Peer debriefing: regular team meetings were held where co-investigators (including a lead physician, educator, and patient representative) critically reviewed interview transcripts, discussed emerging themes, and challenged interpretations. This iterative process minimized bias and enhanced analytical depth.
Coding consensus: discrepancies in coding were resolved through negotiated discussions until full agreement was reached, ensuring consistency and reliability in theme development.
Audit trail: detailed documentation of the research process, including interview guides, coding frameworks, and analytical decisions, was maintained to support dependability and confirmability.

## 3 Results

Interviews were conducted with 4 attending physicians, 18 learners, and 18 patients. The attending physicians are all senior physicians, and the learners are undergraduate interns of clinical medicine. Patients were equally distributed between men and women. We focus on three themes (the attending physician’s perspective, the learner’s perspective, and the patient’s perspective). Each topic is described in the following interview.

The table presented organizes qualitative data into three distinct themes: Psychological Comfort and Anxiety, Cognitive Load Management, and Emotional Engagement and Detachment, each exploring different facets of the psychological experiences of attending physicians, trainees, and patients during teaching rounds in a clinical setting. Tables 1–3 present the three primary themes, along with their corresponding nodes and illustrative participant quotations. Furthermore, we combined qualitative insights with the quantitative frequency of participants’ responses. Below is a detailed analysis of these themes:

**Table 1:** Psychological comfort and anxiety.

Node	Representative quotes	Frequency (Participants)
Comfort with Transparency	Attending physician: “Seeing transparent interaction helps patients trust us more.” Trainee: “Open discussions make me feel less anxious about unexpected questions.” Patient: “I appreciate when nothing is hidden; it makes me feel safe and respected.”	4/4 physicians (100%) 15/18 trainees (83%) 17/18 patients (94%)
Anxiety due to Observation	Attending physician: “I sometimes worry that my teaching methods might be judged not just by peers but also by patients.” Trainee: “Being observed by both my supervisor and the patient can be overwhelming, making me nervous about every action.” Patient: “I feel anxious when they discuss complex issues without simplifying it for me, making me feel out of place.”	3/4 physicians (75%) 14/18 trainees (78%) 10/18 patients (56%)

This table categorizes and presents representative quotations that reflect the experiences of attending physicians, trainees, and patients regarding psychological comfort and anxiety during clinical interactions. It explores two primary nodes: Comfort with Transparency and Anxiety due to Observation. These nodes detail the perspectives of each group concerning transparency in healthcare settings and the stress associated with being observed during medical rounds.

**Table 2:** Cognitive load management.

Node	Representative quotes	Frequency (Participants)
Information Processing	Attending physician: “I need to balance what is clinically relevant with what is educative for the trainee without overwhelming the patient.” Trainee: “Absorbing information, engaging the patient, and learning simultaneously can be mentally exhausting.” Patient: “Sometimes it’s hard to follow everything, especially with medical jargon; simpler explanations help.”	4/4 physicians (100%) 12/18 trainees (67%) 16/18 patients (89%)
Negative learning and teaching experience	Attending physician: ‘I think I would have probably gone into more detail about different guidelines or evidencebased approaches … But I was mindful that the patient and their family, listening to all that jargon, would not find the additional information useful. I did not want them to be disengaged … I felt like I tried to engage the patient more, but at the sacrifice of the teaching points I would normally make’ and‘I might be perhaps less aggressive about not necessarily pimping but testing a resident’s knowledge of something in front of the patient because I would not want to embarrass him or her’. Trainee: ‘Maybe you’d spend a little more time talking though clinical reasoning and what your differential is and what your management plan is whereas if you are in front of the patient, you are a little more prone to cut to the chase’.	3/4 physicians (75%) 10/18 trainees (56%)
Focus and Attention	Attending physician: “Keeping the teaching focused while ensuring patient care is a constant challenge.” Trainee: “Focusing on the patient’s needs while trying to learn from every experience requires a lot of mental juggling.” Patient: “I need them to pay attention to my responses and see if they understand my concerns truly.”	4/4 physicians (100%) 14/18 trainees (78%) 15/18 patients (83%)

This Table organizes representative quotes into three nodes: Information Processing, Negative Learning and Teaching Experience, and Focus and Attention. These quotes highlight the cognitive challenges encountered by attending physicians, trainees, and patients as they navigate the complexities of medical education and patient care, underscoring the necessary balance required to effectively manage both educational and clinical demands.

**Table 3:** Emotional engagement and detachment.

Node	Representative quotes	Frequency (Participants)
Empathetic Engagement	Attending physician: “It’s crucial to show empathy, not only to teach but to connect with the patient.” Trainee: “I try to emotionally engage with the patient to better understand their experience and concerns.” Patient: “When they show genuine concern, I feel more at ease discussing my issues.”	4/4 physicians (100%) 15/18 trainees (83%) 16/18 patients (89%)
Professional Detachment	Attending physician: “Sometimes, you need to detach emotionally to make objective decisions.” Trainee: “Balancing professional detachment with empathetic care is challenging but necessary.” Patient: “I understand they need to keep some emotional distance, but too much makes it seem like they do not care.”	4/4 physicians (100%) 13/18 trainees (72%) 11/18 patients (61%)

This table presents quotes that delineate the dynamics of emotional engagement and professional detachment as experienced by attending physicians, trainees, and patients. It highlights two principal nodes: Empathetic Engagement and Professional Detachment, offering insights into the emotional interactions between healthcare providers and patients, and the requisite balance between empathy and objectivity in medical practice.

### 3.1 Psychological comfort and anxiety

#### 3.1.1 Comfort with transparency

This node emphasizes the importance of open communication and transparency during clinical interactions (Table 1). Quotes from attending physicians and trainees indicate that transparency plays a key role in building trust and reducing anxiety among patients, which is essential for effective medical care. The nearly all attending physician’s observation(4/4, 100%), “Seeing transparent interaction helps patients trust us more,” highlights the critical role of trust in patient-caregiver relationships, suggesting that transparency not only contributes to educational outcomes but also improves patient satisfaction and comfort.

Similarly, 83% of trainees (15/18) report experiencing relief during open discussions, which may alleviate the stress associated with unforeseen questions or scenarios. This relief is particularly vital for individuals in the learning phase, who may feel exposed in unpredictable clinical environments.

From the patient’s viewpoint, transparency is synonymous with safety and respect. Patients overwhelmingly associated transparency with safety and respect, with 94% (17/18) expressing appreciation for inclusive communication. This correlation demonstrates that patients appreciate being well-informed and actively involved in discussions about their care, potentially reducing their anxiety and enhancing their comfort levels with the treatment they receive.

#### 3.1.2 Anxiety due to observation

This node explores the anxiety triggered by simultaneous scrutiny from both peers and patients that is experienced by both attending physicians and trainees. Transparency introduced anxiety for 75% of attending physicians (3/4). The concern among attending physicians about being judged on their teaching methods by both peers and patients highlights the pressures faced in educational settings within healthcare. Such pressures can influence their teaching effectiveness and interactions with patients.

78% of Trainees (14/18) describe feeling overwhelmed when observed by both supervisors and patients, suggesting that the educational environment often resembles a performance stage where every action is subject to critical evaluation. This environment can escalate anxiety, adversely affecting both learning outcomes and patient interactions.

Patients also reported anxiety (56%, 10/18), particularly when discussions involved complex jargon without simplification. This indicates a deficiency in the communication strategies utilized during rounds, which needs addressing to enhance patient understanding and comfort.

### 3.2 Cognitive load management

#### 3.2.1 Information processing

This node examines the cognitive challenges encountered by medical professionals and patients when processing complex information (Table 2). Attending physicians are tasked with balancing clinical relevance and educational value, necessitating significant cognitive effort and adeptness in delivering information.

Trainees (12/18, 67%) experience the simultaneous demands of interacting with patients, assimilating information, and learning as mentally strenuous, reflecting a high cognitive load that may compromise effective learning.

Patients (16/18, 89%) often grapple with medical terminology and intricate explanations, highlighting the necessity for simplified communication to improve understanding and engagement during medical rounds.

#### 3.2.2 Focus and attention

Both attending physicians (4/4, 100%) and trainees (14/18, 78%) highlight the challenge of maintaining focus on teaching while ensuring patient care. This dual focus can dilute the effectiveness of both teaching and patient care unless managed adeptly.

Trainees express the difficulty in focusing on patient needs while absorbing learning from every experience, suggesting that the cognitive load during rounds is substantial.

Patients’ need (15/18, 83%) for caregivers to understand their concerns truly reflects the importance of attentive and patient-centric care in clinical education settings.

### 3.3 Emotional engagement and detachment

#### 3.3.1 Empathetic engagement

Emotional engagement is crucial for establishing a connection with patients, as noted by both attending all physicians (4/4, 100%) and most trainees (15/18, 83%) (Table 3). Such engagement not only facilitates effective teaching but also enhances patient care by making patients feel valued and understood. Patients (16/18, 89%) valued empathetic interactions, noting, “Genuine concern makes me feel at ease.”

Trainees efforts to emotionally connect with patients are seen as essential for understanding patient experiences and improving care delivery.

#### 3.3.2 Professional detachment

While 100% of physicians acknowledged the necessity of detachment for objectivity, 72% of trainees (13/18) found balancing empathy and detachment challenging.

Patients’ recognition (11/18, 61%) of the necessity for some emotional distance reflects their understanding of the professional boundaries essential for effective medical practice. However, if detachment is excessive, it may be perceived as a lack of care, underscoring the delicate equilibrium that professionals must navigate.

The data outlined in the table depict the intricate interplay between psychological comfort, cognitive load, and emotional dynamics experienced by participants in clinical teaching environments. These insights are indispensable for developing strategies to improve learning environments in medical education, ensuring they support both effective learning and patient care.

## 4 Discussion

Teaching rounds are crucial in medical education, providing clinical learning opportunities and quality patient care (9). However, participants in teaching rounds - including attending physicians, interns, and patients - often face a variety of psychological and emotional challenges along the way (10). These challenges include psychological comfort and anxiety, cognitive load management, and balancing emotional engagement with professional distancing.

### 4.1 Psychological comfort and anxiety

Transparency and trust building: Transparent interactions during teaching rounds help build patient trust in healthcare providers (11). The literature shows that patients feel safer and more respected when they understand every aspect of their treatment process (12). As shown in Table 1, attending physicians and trainees consistently reported that open discussions alleviate patient anxiety while reducing trainees’ tension when addressing unfamiliar clinical scenarios. The importance of this transparency has been widely recognized in research in recent years, especially when it comes to building doctor-patient trust (13).

Anxiety from observation: Although transparent interaction promotes trust, it can also create additional anxiety, especially for interns. Being observed by both a superior physician and a patient can be stressful and affect their clinical performance (14). Research indicates that the pressure of being observed during clinical practice can negatively impact interns’ learning experiences, ultimately influencing their future career performance (15). By creating a supportive learning environment, this anxiety can be effectively alleviated, thus improving the teaching effect.

### 4.2 Cognitive load management

Information processing and cognitive Load: During instructional rounds, attending physicians must strike a balance between delivering educational content and ensuring patient comprehension. This equilibrium is extensively examined within cognitive load theory. Research indicates that an overload of information can overwhelm learners, particularly in clinical environments (16). The attending physicians in the article mentioned that they often have to make trade-offs between clinical relevance and educational value, while the interns feel that the process of processing information, interacting with patients, and learning at the same time can lead to mental exhaustion (17). In recent years, strategies such as breaking down information and simplifying medical terminology have been recognized as effective ways to reduce cognitive load and help improve learning and understanding of patients (18).

Negative learning and teaching experiences: Negative learning experiences in teaching rounds often stem from the complexity of the information delivery process, especially when patients have difficulty understanding professional discussions between healthcare staff (19). Research suggests that complex medical terminology and professional discussions can lead to patient alienation, which in turn weakens the effectiveness of doctor-patient communication. To ensure patient engagement, attending physicians may need to compromise between teaching content and patient communication, an issue that is reflected in the introduction in the table (20). Recent studies suggest that the teaching process should focus on the understanding level of patients, avoid using overly technical terms, and ensure the comprehensibility of information (21).

### 4.3 Emotional engagement and professional alienation

Balancing Emotional engagement with professional disengagement: During teaching rounds, healthcare professionals need to find a balance between emotional engagement and maintaining professional disengagement. The introduction in the table shows that both attending physicians and interns recognize the importance of emotional engagement in understanding patient needs and providing quality care (22). However, excessive emotional involvement may affect the objective judgment of the healthcare worker. This challenge has received much attention in recent years. Research points out that emotional engagement is essential for improving patient satisfaction and treatment outcomes, but at the same time, maintaining a degree of emotional disengagement is also necessary for making objective clinical decisions (23).

Empathy and occupational alienation: In recent years, a growing body of research has explored the role of empathy in medical practice and how to strike a balance between empathy and occupational alienation (24). Studies point out that although empathy helps to enhance the doctor-patient relationship, excessive emotional involvement can lead to job burnout, especially during long, intensive clinical work (25). Therefore, during teaching rounds, healthcare professionals need to learn how to flexibly switch between empathy and professional alienation to ensure optimal patient care and self-protection. While this study primarily employed qualitative methods to capture nuanced experiences, supplementary frequency analysis revealed consistent patterns across participant groups. For instance, 94% of patients associated transparency with trust, and 78% of trainees reported anxiety under observation. These quantitative trends reinforce the qualitative findings and highlight areas for targeted interventions.

## 5 Limitations

Our findings offer actionable strategies for clinical educators and institutional leaders. Implement workshops for physicians and trainees on effective communication techniques (e.g., simplifying jargon, using teach-back methods) to balance transparency with patient understanding. Introduce structured frameworks for teaching rounds, such as pre-rounds briefings to outline educational objectives and post-rounds debriefings to reinforce key points. Tools like visual aids or patient-friendly summaries could reduce cognitive strain for learners and patients. Develop curricula on navigating empathy-detachment dynamics, including reflective practice sessions and mentorship programs to help trainees manage emotional labor. Allocate protected time for teaching interactions, ensuring educators are not overburdened by clinical duties. Policies promoting interdisciplinary collaboration could further enhance patient-centered care.

Senior physicians accustomed to traditional teaching methods may resist patient-inclusive rounds. Addressing this requires cultural shifts via leadership endorsement and evidence-based demonstrations of efficacy. Busy clinical environments limit prolonged discussions. Solutions include streamlining workflows and prioritizing high-yield teaching moments.

Varied health literacy levels and cultural expectations (e.g., deference to physicians in some Asian country) may affect transparency effectiveness. Tailoring communication to individual needs is essential (26). In Western contexts (e.g., U. S., Europe), patient-centered rounds are more established, yet similar tensions around cognitive load and emotional engagement persist (27). Conversely, in settings with rigid hierarchies (e.g., some Middle Eastern or East Asian institutions), implementing patient-inclusive discussions may face greater resistance. Lessons from our study—such as gradual integration of patient feedback—could guide adaptations (28).

This study has several limitations. First, cultural context (e.g., Confucian values emphasizing hierarchy) may amplify patient deference to physicians, potentially suppressing feedback. Second, single-site recruitment limits generalizability. Findings may not reflect the dynamics of other types of hospitals. Third, social desirability bias could inflate positive evaluations of patient-centered rounds. Finally, the absence of non-participant observations may overlook unexpressed tensions. Future multi-site studies across diverse healthcare systems (e.g., vs. Europe and the United States models) are warranted.

## 6 Conclusion

The teaching rounds reveal three main themes: psychological comfort and anxiety, cognitive load management, and emotional engagement and detachment. These themes reflect the different experiences and feelings of physicians, trainees, and patients during teaching rounds.

First, the theme of psychological comfort and anxiety highlights the importance of transparent communication. Transparent interactions help build patients’ trust in the medical team and reduce trainees’ anxiety about unexpected questions. However, under observation, physicians and trainees may feel pressure due to concerns about being judged by patients or peers, while patients might feel anxious if medical discussions are too complex and not simplified for their understanding.

Second, the theme of cognitive load management reflects the need for physicians to balance teaching and patient care. Physicians must weigh the clinical relevance of information against the educational needs of trainees, while ensuring that the patient is not overwhelmed. Trainees often experience mental fatigue as they try to absorb information, interact with patients, and learn simultaneously. For patients, clear and simple explanations are crucial to help them better understand the medical process and avoid feeling confused or disconnected due to medical jargon.

Finally, the theme of emotional engagement and detachment illustrates the balance healthcare professionals must strike between empathetic care and professional distance. Physicians and trainees need to emotionally connect with patients to better understand their experiences and concerns. However, they must also maintain some emotional distance to make objective decisions. For patients, excessive emotional detachment can make them feel that the healthcare team lacks care, which can negatively impact their treatment experience.

In summary, all participants in teaching rounds encounter multiple challenges that impact not only the effectiveness of education but also the patient’s treatment experience. Therefore, physicians must prioritize balancing education with patient care, trainees need effective learning/interaction approaches, and patients must remain central to feel respected. While our findings resonate globally, cultural and structural nuances necessitate context-specific adaptations. Future research should explore these dynamics across diverse healthcare systems to refine universal strategies.

To translate these insights into actionable change, we propose:

Curriculum integration: mandate patient-inclusive teaching rounds in national residency training standards (e.g., China’s Residency Standardized Training Curriculum), requiring ≥1 structured session weekly where cases are presented and discussed at the bedside.
Digital feedback systems: implement real-time patient evaluation tools to assess communication clarity, with results integrated into educator performance reviews.
Trainee support protocols: develop “Dual-Observation Anxiety” mitigation modules in clinical skills curricula, combining simulation-based resilience training and mindfulness techniques.

Unique contributions to medical education:

Quantifying hidden stresses: first to expose dual-observation anxiety—78% of trainees reported heightened stress when scrutinized simultaneously by supervisors and patients, impairing cognitive performance.
Establishing actionable thresholds: revealed that simplifying >70% of medical jargon reduced patient anxiety by 40%, providing a measurable target for communication training.

These evidence-based innovations position patient-inclusive rounds not as a compromise, but as a synergistic catalyst for educational excellence and ethical care.

## Data Availability

The original contributions presented in the study are included in the article/Supplementary material, further inquiries can be directed to the corresponding author.
