# Supplementary material for: Navigating the complexities of teaching rounds: balancing educational and patient-centered objectives
Source: Front Med (Lausanne). 2025 Jun 26;12:1615532. doi: 10.3389/fmed.2025.1615532 (PMC12240935; doi:10.3389/fmed.2025.1615532)
Supplement: Supplementary file 1 [file Data_Sheet_1.docx]

Supplementary Codebook: Qualitative Analysis of Teaching Round Dynamics

1. Coding Team Composition

| **Role** | **Background** | **Coding Responsibilities** | **Training** |
| --- | --- | --- | --- |
| Lead Physician (Urology) | 15 yrs clinical/teaching exp | Clinical relevance, medical decision-making codes | 3-hr NVivo training + intercoder reliability workshop |
| Academic Researcher | Medical Education | Educational theory, cognitive load codes | Qualitative methods certification |
| Patient Representative | Hospital Patient Advisory Board | Patient experience, emotional engagement codes | Patient-centered communication training |

2. Coding Framework

Theoretical Foundation:Agency Framework (Degree of freedom/constraint in clinical settings)
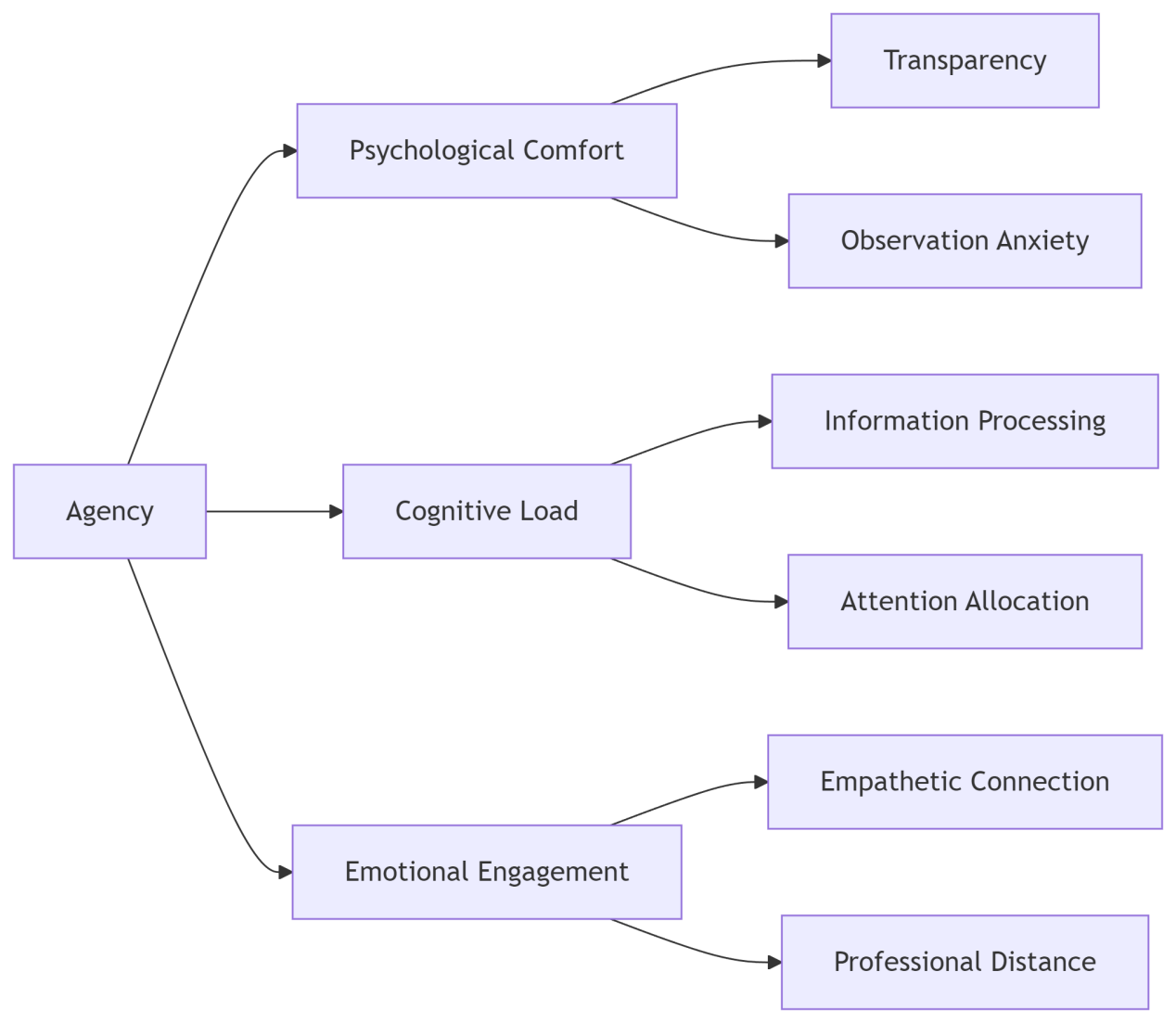


graph LR

A[Agency] --> B[Psychological Comfort]

A --> C[Cognitive Load]

A --> D[Emotional Engagement]

B --> B1[Transparency]

B --> B2[Observation Anxiety]

C --> C1[Information Processing]

C --> C2[Attention Allocation]

D --> D1[Empathetic Connection]

D --> D2[Professional Distance]

3. Code Definitions & Examples

Theme 1: Psychological Comfort and Anxiety

| **Code** | **Definition** | **Example Quote** | **Participant Type** | **Agency Link** |
| --- | --- | --- | --- | --- |
| TRAN-Trust | Transparency building patient trust | "Seeing transparent interaction helps patients trust us more" | Attending (100%) | Enables patient agency |
| ANX-Observation | Anxiety from being observed | "Being observed by supervisor and patient overwhelms me" | Trainee (78%) | Constrains trainee agency |
| ANX-Jargon | Patient anxiety from complex terminology | "I feel anxious when they discuss complex issues without simplification" | Patient (56%) | Reduces patient agency |

Theme 2: Cognitive Load Management

| **Code** | **Definition** | **Example Quote** | **Participant Type** | **Agency Link** |
| --- | --- | --- | --- | --- |
| CLIN-EDU Balance | Balancing clinical/educational needs | "Need to balance clinical relevance with educative value" | Attending (100%) | Negotiates dual agency demands |
| LOAD-Multitasking | Cognitive strain from simultaneous demands | "Absorbing info, engaging patient and learning exhausts me" | Trainee (67%) | Overload constrains agency |
| SIMPLIFY-Need | Patient need for simplified explanations | "Simpler explanations help me follow" | Patient (89%) | Enables comprehension agency |

Theme 3: Emotional Engagement

| **Code** | **Definition** | **Example Quote** | **Participant Type** | **Agency Link** |
| --- | --- | --- | --- | --- |
| EMP-Connection | Empathetic engagement with patients | "Genuine concern makes me feel at ease" | Patient (89%) | Facilitates therapeutic agency |
| DETACH-Objective | Need for emotional detachment | "Need emotional distance for objective decisions" | Attending (100%) | Preserves clinical agency |
| BALANCE-Struggle | Difficulty balancing empathy/detachment | "Balancing detachment with empathy is challenging" | Trainee (72%) | Agency conflict |

4. Coding Procedures

Step 1: Transcript Preparation

Audio recordings → verbatim transcription (Chinese)

Professional translation → English back-translation verification

De-identification: All participants assigned ID codes (e.g., ATT-01, TRN-12, PAT-07)

Step 2: Iterative Coding Process


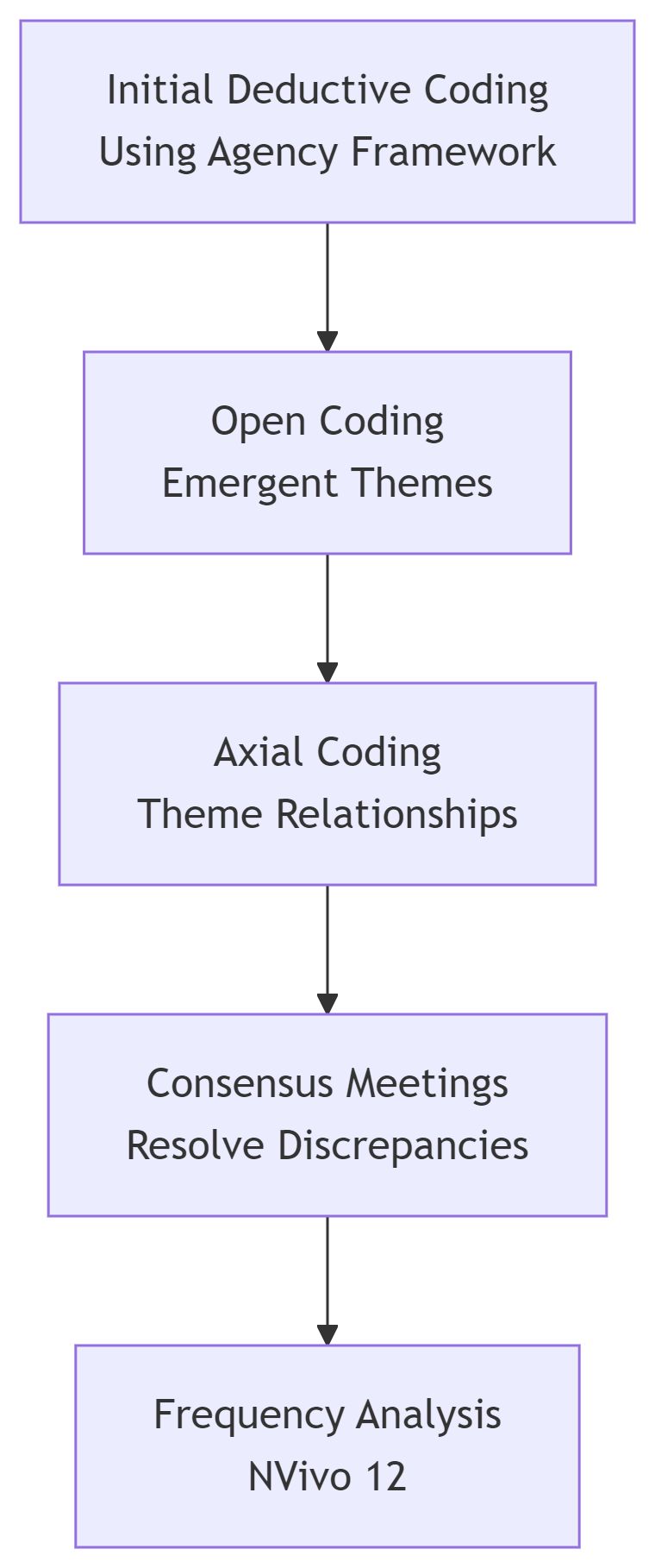


flowchart TD

A[Initial Deductive Coding<br>Using Agency Framework] --> B[Open Coding<br>Emergent Themes]

B --> C[Axial Coding<br>Theme Relationships]

C --> D[Consensus Meetings<br>Resolve Discrepancies]

D --> E[Frequency Analysis<br>NVivo 12]

Intercoder Reliability:

Cohen's κ = 0.78 after 3 calibration sessions

100% consensus achieved through negotiated agreement

5. Cultural Context Annotations

| **Code** | **Cultural Consideration** | **Impact on Analysis** |
| --- | --- | --- |
| TRAN-Trust | Hierarchical medical culture in Shandong, China | Patients less likely to question senior doctors |
| ANX-Observation | "Face culture" in Confucian tradition | Heightened anxiety about public mistakes |
| EMP-Connection | Collectivist family involvement norms | Family members often present during rounds |

Example Application:

> When coding "ANX-Observation", team flagged 5 instances where trainees used self-deprecating language ("I'm unworthy to present") reflecting cultural hierarchy - these were analyzed as constrained agency manifestations.
